# Nuclear and peroxisomal targeting of catalase

**DOI:** 10.1111/pce.14262

**Published:** 2022-01-27

**Authors:** Yousef Al‐Hajaya, Barbara Karpinska, Christine H. Foyer, Alison Baker

**Affiliations:** ^1^ Centre for Plant Sciences and School of Molecular and Cellular Biology University of Leeds Leeds UK; ^2^ Centre for Plant Sciences and School of Biology University of Leeds Leeds UK; ^3^ Astbury Centre for Structural Molecular Biology University of Leeds Leeds UK; ^4^ Present address: Department of Laboratory Medical Sciences Mutah University Karak Jordan; ^5^ Present address: School of Biosciences, College of Life and Environmental Sciences University of Birmingham Edgbaston UK

**Keywords:** nucleus, peroxisome, redox signalling, ROS

## Abstract

Catalase is a well‐known component of the cellular antioxidant network, but there have been conflicting conclusions reached regarding the nature of its peroxisome targeting signal. It has also been reported that catalase can be hijacked to the nucleus by effector proteins of plant pathogens. Using a physiologically relevant system where native untagged catalase variants are expressed in a *cat2‐1* mutant background, the C terminal most 18 amino acids could be deleted without affecting activity, peroxisomal targeting or ability to complement multiple phenotypes of the *cat2‐1* mutant. In contrast, converting the native C terminal tripeptide PSI to the canonical PTS1 sequence ARL resulted in lower catalase specific activity. Localisation experiments using split superfolder green fluorescent protein revealed that catalase can be targeted to the nucleus in the absence of any pathogen effectors, and that C terminal tagging in combination with alterations of the native C terminus can interfere with nuclear localisation. These findings provide fundamental new insights into catalase targeting and pave the way for exploration of the mechanism of catalase targeting to the nucleus and its role in non‐infected plants.

## Summary statement

*Arabidopsis thaliana* CAT2 is targeted to the nucleus and the peroxisome. Peroxisome targeting was independent of the C terminus and required the noncanonical import pathway for optimal activity. Modification to the C terminus interfered with nuclear targeting in the absence of pathogen effectors.

## 1 INTRODUCTION

Reactive oxygen species (ROS) are major products of plant energy metabolism in mitochondria, chloroplasts and peroxisomes (Dietz et al., 2016; Huang et al., 2016; Sandalio & Romero‐Puertas, 2015). They are also ubiquitous signalling molecules in plants and animals. ROS play a central role in plant growth and defence, with a convergence of host and pathogen proteins on ROS scavenging to regulate immunity and growth. A major component of this interaction is the extracellular production of ROS by the conserved NADPH oxidase (Rboh) family that regulate immune functions, cell growth, and apoptosis in animals and plants (Waszczak et al., 2018). RBOH‐dependent ROS production is a common response to the activation of receptor‐like protein kinase (RLK) signalling, in particular following perception of microbe‐associated molecular patterns (MAMPs) or damage‐associated molecular patterns (DAMPs), a process that has been suggested to involve the hydrogen peroxide (H_2_O_2_)‐scavenging enzyme, catalase (CAT). CAT is a target for plant pathogen‐encoded effectors, which traffic CAT to the nucleus to regulate programmed cell death (Inaba et al., 2011; M. Zhang et al., 2015).

CAT is predomantly localised in peroxisomes, where it accounts for 10%–25% of peroxisomal protein (Reumann et al., 2004). This tetrameric heme‐containing enzyme catalyses the dismutation of H_2_O_2_ to water and oxygen (Gechev et al., 2006). CAT is encoded by a small gene family in *Arabidopsis thaliana*. The CAT2 protein is the major leaf isoform and plays a crucial role in photorespiration (reviewed in Mhamdi et al., 2012). Like all peroxisomal proteins, CAT is synthesised in the cytoplasm and directed to the peroxisome. Targeting of peroxisomal matrix proteins utilises two types of peroxisomal targeting signals, PTS1 and PTS2, which are recognised by cytoplasmic receptors PEX5 and PEX7, respectively (Gould et al., 1989; Kato et al., 1996; Nito et al., 2002). Recently, a third type of PTS targeting signal has been identified which is known as PTS3. Unlike the linear PTS1 and PTS2, the PTS3 is a signal patch (Kempiński et al., 2020). Non‐PTS proteins are also able to import into peroxisomes by a mechanism called piggy‐back import (Glover et al., 1994; Lee et al., 1997).

In general, the C‐terminus tripeptide consensus sequence of PTS1 is (S/A/C)‐(K/R/H)‐L (Brocard & Hartig, 2006) which is obligatorily at the C terminus of the protein and binds within a funnel‐shaped binding pocket of the C terminal TPR domain of PEX5 (Gatto et al., 2003; Stanley et al., 2006) but the C‐terminus of CAT2 (which is PSI) does not fit this consensus (reviewed in Mhamdi et al., 2012). Several studies have addressed the peroxisomal targeting of plant CAT. For example, the last four amino acids of cottonseed CAT (Ccat) were required for targeting of a reporter protein to tobacco BY‐2 suspension culture cells (Mullen et al., 1997). In contrast, the last 10 amino acids of the pumpkin CAT were not required for the peroxisomal localisation of green fluorescent protein (GFP) in stably transformed BY‐2 cells (Kamigaki et al., 2003). Other mutations elsewhere in the protein also prevented import (Fujikawa et al., 2019).

In this study, we have reinvestigated the requirements for targeting the *A. thaliana* CAT2 (*At*CAT2). We identified an alternative splice variant of CAT2 gene in the TAIR database (At4g35090.2). This variant arises from non‐splicing of the last intron, which removes the last 18 amino acids of the CAT protein. Using a combination of physiological, biochemical and cell biology approches, we analysed the effects of introducing *At*CAT2 variants with modified C‐termini into the *cat2‐1* mutant background. The comprehensive evaluation of activity, function and targeting in a physiologically relevant context revealed that the last 18 amino acids of the CAT2 sequence are dispensable for growth, cellular redox status and targeting of the enzyme to the peroxisomes, but alteration of this region interferes with nuclear localisation.

## 2 MATERIALS AND METHODS

### 2.1 Preparation of constructs

To create the transgenic lines CAT2^PSI^, CAT2^WSQV^ and CAT2 ^ARL^ under the control of the CAT2 promoter, 2.4 kb genomic DNA upstream of the CAT2 gene was amplified with primers Cat2promR and Cat2prom F (all primers are listed in Table S1). The Cat2promR contains restriction sites *Bsr*GI and *Xba*I. The sequence of the potential promoter region also contains *Hin*dIII at the 5′ end. The PCR product was cut with *Xba*I and *Hind*III and cloned into pGreen0179 +NosT to generate cat2prom‐nos. CAT2 variants corresponding to CAT2^PSI^ CAT2^WSQV^ and CAT2^ARL^ were amplified from Arabidopsis leaf and seedling cDNA using the primer CAT2F in combination with CAT2RN, CAT2RWSQV and CAT2RARL. Correct sized amplification products were cloned into pDONOR207 by BP reaction and verified by restriction digest and sequencing. CAT2 variants were excised as *Bam*HI and *Bsr*GI fragments and cloned into *Bam*HI and *Bsr*GI digested cat2prom‐nos. Finally, the native 3′UTR was placed in front of the Nos terminator. Primers PSI‐3UTR‐F and PSI‐3UTR‐R and WSQV‐3UTR‐F and ARL‐3UTR‐R were used to amplify the sequence immediately 3′ to the stop codon of the normal and splice variant transcript and to introduce flanking *Bsr*GI and *Xba*I sites. PCR products and cat2promnos plasmids containing the CAT2 variants were digested with *Bsr*GI and *Xba*I and ligated together to produce plasmids cat2promoter‐CAT2‐PSI‐3UTR‐nos, cat2promoter‐CAT2‐WSQV‐3UTR‐nos and cat2promoter‐CAT2‐ARL‐3UTR‐nos (Figure S1). These plasmids were transformed into the *cat2‐1* mutant background by floral dip (Clough & Bent, 1998). Transformants were selected on hygromycin and three independent homozygous lines of CAT2^WSQV^ and CAT2^PSI^ and two independent homozygous lines of CAT2^ARL^ were used in this study. To prepare C terminal SfGFP constructs, plasmids cat2promoter‐CAT2‐PSI‐3UTR‐nos, cat2promoter‐CAT2‐WSQV‐3UTR‐nos and cat2promoter‐CAT2‐ARL‐3UTR‐nos were used as a PCR template with CAT2PSIF, CAT2PSIR, CAT2WSQVR and CAT2ARLR primers. These PCR products contain *KpnI* and *SpeI* restriction sites. After gel purification and digestion with *Kpn* I and *Spe*l they were ligated into *KpnI* and *SpeI* digested PEP109E plasmid (Park et al., 2017). To prepare N terminal sfGFP plasmids cat2promoter‐CAT2‐PSI‐3UTR‐nos, cat2promoter‐CAT2‐WSQV‐3UTR‐nos and cat2promoter‐CAT2‐ARL‐3UTR‐nos were digested with *Bam*HI and *Kpn*I and ligated with a synthetic fragment comprising the UBQ10 promoter sequence from PEP109E, The GFP11 coding sequence and the start of the CAT2 gene from *Bam*HI and *Kpn*I digested pUBQ10GFP11Cat‐pUC57 (Figure S2).

#### 2.1.1 Plant materials and growth conditions

*Arabidopsis thaliana* wild type lines (Col‐0) and the *cat2‐1* mutant (Queval et al., 2007) were obtained from the Nottingham Arabidopsis Centre. Transgenic *A. thaliana* lines that express sfGFP1‐10^OPT^ targeted to peroxisomes, nucleus and cytoplasm generated by Park et al. (2017) were obtained from Arabidopsis Biological Resource Centre (ABRC).

Seeds were sown on ½ MS media (with 50 µg/ml hygromycin for transgenic lines) and stratified for 2 days in the dark at 4°C followed by 6 h at 20°C in continuous white light (150–190 µmol m^−2^ s^−1^). The plates were then wrapped in aluminium foil for 2 days at room temperature, the foil was removed, and plates were placed for 5 days in 16 h light. Then, seedlings were transferred into soil and grown 4 weeks in a controlled environment growth chamber (8 h light, 20°C, humidity 60%). before transfer to long day conditions (16 h light 21°C, humidity 60%) for further 1 week. All samples were rapidly frozen in liquid nitrogen and stored at −80°C for subsequent analysis. All samples were taken 4–4.5 h into the photoperiod. The photorespiration‐promoting conditions were applied according to the protocol described by Waszczak et al. (2016). Arabidopsis protoplasts were prepared from leaves of 5‐ to 6‐week‐old plants grown under controlled conditions (Light intensity: 180 µmol. m^−2^. s^−1^, 16 h light and 8 h dark, 22°C) and transfected according to Wu et al. (2009).

#### 2.1.2 Quantitative real‐time PCR analysis

Total RNA was extracted from leaves of 4‐week‐old plants with RNeasy plant mini kit (QIAGEN) and treated with TURBO DNA‐free kit (Invitrogen) according to the manufacturer's instructions. The first‐strand cDNA was synthesised using QuantiTect reverse transcription kit (QIAGEN). Real‐time PCR reactions were assayed using the Brilliant ®II SYBR® Green Q PCR master Mix (Agilent, CAT#600828‐51) on a CFX connect real‐time PCR system (Bio‐Rad Laboratories). All transcripts were normalised to *ACTIN2*. Specific primers used for quantification are listed in (Table S1). AGI codes for the gene encoding marker transcripts: At2g29500 (*HSP17.6*). *GSTF8*, At2g47730. At3g25250, OXI 1. The expression level was calculated using the 2^‐ΔΔCT^ method.

#### 2.1.3 Glutathione and ascorbate quantifications

Glutathione and ascorbate were assayed as described (Noctor et al., 2016). Briefly, 100 mg of frozen leaves was ground in 1 ml perchloric acid (HClO_4_). The homogenate was centrifuged at 14 000 rpm at 4°C and the supernatant was adjusted to pH 6.0. Glutathione and ascorbate were quantified using Greiner F‐bottom 96‐well UV‐transparent plates on a FLUOstar Omega microplate reader (BMG Labtech).

#### 2.1.4 Enzyme activity measurements

Approximately 100 mg leaf material was ground in liquid nitrogen. 1.5 ml 0.1 M NaH_2_PO_4_ (pH 7.5), 1 mM EDTA was added to the extract. The homogenates were centrifuged for 10 min at 14 000 rpm at 4°C, and the supernatants were used for the assay. Catalase activity assay was performed according to Veljovic‐Jovanovic et al. (2001). Hydroxy pyruvate reductase (HPR) activity was measured by monitoring the NADH oxidation at 340 nm. The reaction mixture (1 ml total volume) contained 200 mM KH_2_PO_4_/K_2_HPO_4_ (pH 6.7), 2.8 mM NADH, 100 mM HPR and plant extract). Protein was assayed using BCA method (Thermo Scientific, Pierce™ BCA protein assay reagent A and B with prod # 23223 and 23224, respectively). Chlorophyll quantification was determined by absorbance of the samples was measured at 646.6, 663.6 and 750 nm in a glass cuvette and calculated according to the extinction coefficients described in Porra et al. (1989).

#### 2.1.5 Western blot analysis

Proteins were separated by 12% SDS‐PAGE and electroblotted onto nitrocellulose membranes, blocked in 5% (w/v) low fat dried milk in Tris‐Buffered with Tween (20 mM Tris‐HCl, 150 mM NaCl, 0.1% [v/v] Tween 20 pH 7.6) 1 h at room temperature (RT) with shaking. The membranes were incubated with primary antibodies (Anti‐CAT [1:1000; Agrisera; AS09501], Anti‐CAT2 [1:20,000], [Su et al., 2018], Anti‐AtpB [1:5000; Agrisera; AS05085‐10]) overnight at 4°C with agitation. The primary antibodies were decanted, and the blots were washed 3 × 10 min with TBS‐T at RT followed by Goat anti‐rabbit (1:5000, HRP; Jackson ImmunoResearch Europe LTD; 111‐035.003) for anti‐CAT and anti‐AtpB, Goat anti‐mouse IgG H&L (1:10 000; Abcam; ab6789) for anti‐CAT2 for 1 h at RT with shaking. The blots were washed 3 × 10 min with TBS‐T and developed in a dark room using chemilumiescence substrate (super signal west Dura, USA).

#### 2.1.6 Gel analysis and activity assay

One hundred milligram leaves of 4‐weeks plants were ground to a powder in liquid nitrogen and then homogenised in native extraction buffer (100 mM Tris‐HCl, pH 8.0, 20% glycerol and 30 mM dithiothreitol [DTT]). After centrifugation at 14 000 *g* for 30 min at 4°C, the supernatant was recovered and 20 µg of total protein was separated by 7.5% native gel (Bio‐Rad) 5 h (70 V) at room temperature in electrophoresis buffer (192 mM glycine, 25 mM Tris‐HCl, pH 8.3). Analysis of catalase using in‐gel activity assay was carried out by using the protocol described in Weydert and Cullen (2010).

### 2.2 Isolation of leaf peroxisomes

For crude fractionation, 2 g of 4‐week‐old wild type, *cat2‐1*, CAT2^PSI^, CAT2^WSQV^ and CAT2^ARL^ leaves were harvested and homogenised in extraction buffer (Tricine [170 mM], KCl [10 mM], MgCl_2_ [1 mM], EDTA [1 mM], Sucrose [1 mM] and DTT [5 mM] pH 7) using a parsley chopper. Following cell disruption, three fractions were prepared as follows: The first fraction, the homogenate fraction (S1 supernatant), was obtained by centrifugation of the homogenate (10 min 2000 *g*). The second fraction, the cytosolic fraction (S2), was obtained by centrifugation of 1 ml of the S1 supernatant (20 min 12 000 *g*). Finally, the S2 pellet (organelle fraction) was suspended in 0.1 ml of the homogenisation buffer. For pure peroxisome isolation, 3–5 weeks‐old wild type, *cat2‐1* mutant, CAT2^PSI^, CAT2 and CAT2^ARL^ leaves (5–10 *g*) were harvested and leaf peroxisomes were isolated as described previously (Reumann et al., 2007). The isolation procedure was performed in a cold‐room (4°C).

### 2.3 Confocal microscopy analysis

Protoplasts were observed with a Zeiss 700 laser scanning confocal microscopy using EC Plan‐Neofluar 20x/0.5 M27. Excitation wavelengths and emission filters were 8.5% of 488‐nm diode laser/band‐pass 300–531 nm to detect sfGFP. Negative controls were carried out to check that there was no bleed through of fluorescence from the chlorophyll channel.

## 3 RESULTS

### 3.1 C‐terminal modifications of *At*CAT2 complement the *cat2‐1* mutant for growth

**Figure 1:**
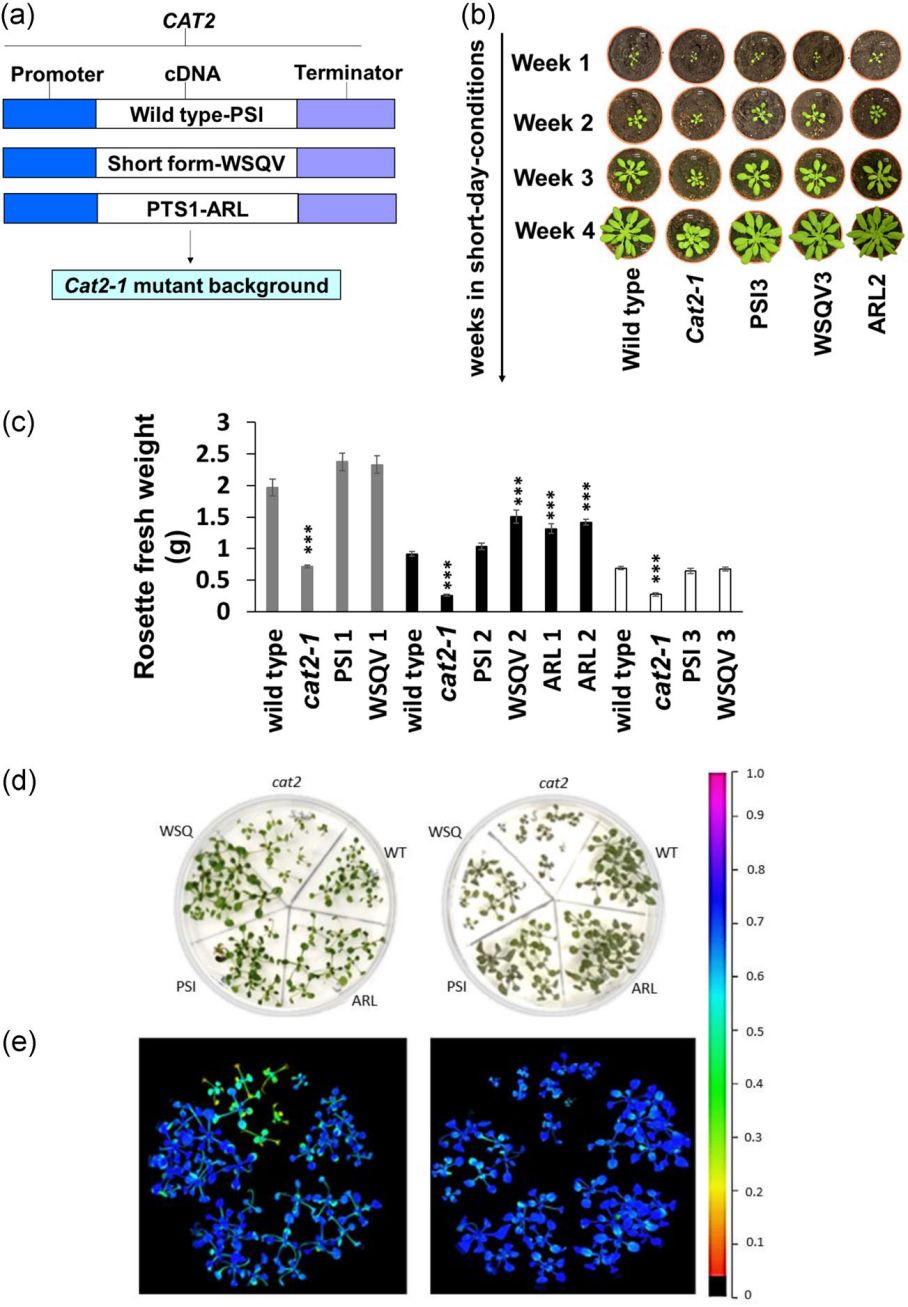
Catalase C terminal variants rescue growth phenotypes of the *cat2‐1* mutant in normal air and under photorespiratory conditions. (a) Transgenic lines were generated by expressing different variants of CAT2 under its own promoter and 3′UTR. (b) 2‐ 3‐ 4‐, and 5‐week‐old plants of wild type, *cat2‐1*, 3rd independent line of PSI3 and WSQV3, and 2nd independent line of ARL2 grown under short‐day conditions (Light intensity: 190 µmol. m^−2^. s^−1^, 8 h light and 16 h dark, 20°C, 60% humidity) for 4 weeks. Scale bars represent 1 cm. All plants photographed at the same magnification once a week for 4 weeks. Plants photographed at the 1st, 2nd, 3rd and 4th weeks (indicated as Week 1, Week 2, Week 3 and Week 4, respectively). The experiment was repeated three times (three independent lines) with similar results. (c) Rosette fresh weight; three independent lines are presented (grey, black and white bars). With each independent line, wild type and *cat2‐1* mutant were also grown under identical growth conditions. Plants presented by black and white bars are 5‐week‐old. In comparison, plants presented by grey bars are 6 weeks old. Means were calculated from six plants per experiment. *** Significant difference from wild type at *p* < 0.001 by Student *t*‐test. Error bars represent the SE. (d) Phenotypes of wild type, *cat2‐1*, and transgenic lines under photorespiratory and control conditions. The appearance of plants grown at 16/8 h day/night photoperiod at 22°C for 2 weeks and subjected to 100 µmol m^−2^ s^−1^ constant light for 1 week with air‐tight tape (left image) and air‐permeable tape (right image). (e) False‐colour images of Fv/Fm (maximum quantum yield of photosystem II) [Color figure can be viewed at wileyonlinelibrary.com]

Previous studies have yielded contradictory results regarding the precise molecular determinants of catalase targeting, particularly the role of the carboxy‐terminal amino acids. We therefore addressed this question in a physiologically relevant context where catalase activity and targeting were evaluated using the following constructs: the wild type CAT2 sequence (terminating PSI), a C terminal truncation arising from the retention of an intron that replaces the last 19 amino acids with a valine (terminating WSQV) and a variant in which the C terminal tripeptide was mutated to PTS1 consensus sequence (terminating ARL). Intron retention is the most common form of alternative splicing event (R. Zhang et al., 2017). This variant is detectable by PCR on polysomal RNA (Figure S3) so is presumably translated but is present at very low levels in published transcriptome data sets (Calixto et al., 2018; R. Zhang et al., 2017). These constructs were expressed under the control of the native CAT2 promoter with the native 3′UTR in the *cat2‐1* mutant background (Queval et al., 2007) (Figure 1a). In these experiments, the transgenic lines (three independent lines for PSI and WSQV and two independent lines for ARL) were grown together with the wild type and the *cat2‐1* mutant for up to 5 weeks under short‐day growth conditions before transfer to long‐day growth conditions for a further 1 week (Figure S4 for images of all independent transgenic lines). The *cat2‐1* mutant rosettes were visibly smaller than the wild type under this growth regime, as previously reported (Queval et al., 2007). However, all the lines expressing all the variants had comparable growth and development to the wild type (Figure 1b) and rosette fresh weight was restored (Figure 1c). The leaf number and rosette diameter confirmed that the expression of all variants complemented the wild type growth phenotype (Figures S5 and S6). The primary root length was decreased in the *cat2‐1* mutant, but root length was fully restored to wild type levels in all of the transgenic lines (Figure S7).

CAT2 is a key enzyme of the photorespiratory pathway in leaves. Flux through the photorespiratory pathway was increased by growing plants under continuous light in conditions where gas exchange with the environment was restricted (Waszczak et al., 2016). Under the enhanced photorespiratory conditions, the *cat2‐1* mutants showed visible symptoms of chlorosis (Figure 1d). In contrast, the wild type, and complemented lines had no visible symptoms. These observations were confirmed by Fv/Fm measurements (Figure 1e).

### 3.2 *At*CAT2 with C‐terminal modifications are enzymatically active and assemble correctly

**Figure 2:**
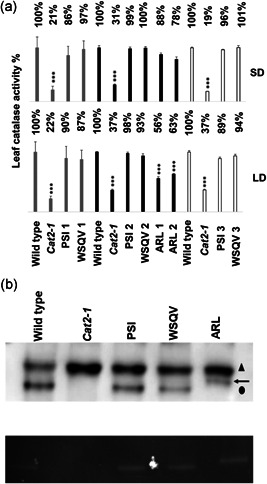
Restoration of catalase activity in transgenic lines and variants form active homo‐tetramers. (a) Catalase activity was measured in leaf extracts from wild type, *cat2‐1* mutant and different independent transgenic lines grown under short (8hL 16hD) and long‐day (16hL 8hD) conditions and sampled at the same time relative to start of the light period. For each line, three biological replicates were each measured in triplicate. Grey, black and white bars are data from separate experiments. Percentage of catalase activity relative to wild type in each experiment is indicated at the top of the graph (activities are given in Table S2). Error bars represent the SE. *** Indicates significantly lower catalase activity (*p* < 0.001) by Student's *t‐*test compared to the wild type in each experiment. (b) Characterisation of catalase isoforms by native PAGE. Upper panel: Total protein (20 µg per line) extracted from leaves of 4‐week‐old wild type, *cat2‐1*, CAT2^PSI^, CAT2^WSQV^ and CAT2^ARL^ was separated on native‐PAGE and blotted on nitrocellulose membrane with catalase antibody (Agrisera, AS09501) which recognises all three catalase isoforms (CAT1, CAT2 and CAT3). Cross reacting protein (triangle) and CAT2 (circle). A shifted band of CAT2^ARL^ is indicated by an arrow. Lower panel: in‐gel activity assay

The ability of all of the expressed CAT2 variants to restore the growth phenotype of the *cat2‐1* mutants suggests that expression of all the sequences leads to the production a functional enzyme. Leaf CAT activity was measured in three independent transgenic lines of CAT2^PSI^ and CAT2^WSQV^ and two independent transgenic lines of CAT2^ARL^ that had been grown under both short‐ and long‐day conditions. CAT activities are shown in (Table S2). To enable comparisons of data from independent experiments (grey, black and white bars in Figure 2a) activities are expressed relative to the wild type, for which values were set at 100% in each experiment. Leaf CAT activities were similar in all lines grown under short‐day conditions (Figure 2a, SD). The CAT activities of the wild type, CAT2^PSI^ and CAT2^WSQV^ lines were also similar in plants grown under long‐day conditions but the CAT2^ARL^ lines had a significantly lower CAT activity than the other lines (Figure 2a, LD).

These findings suggest that CAT is correctly assembled into tetramers in the CAT2^PSI^, CAT2^ARL^ and CAT2^WSQV^ lines. To confirm this possibility and to investigate whether mixed tetramers of CAT2 with CAT1 or CAT3 were formed, leaf extracts were subjected to native PAGE, western blot analysis and in‐gel activity assays. A single immune‐reactive band was clearly detected in wild type leaves, whereas this band was absent from the *cat2‐1* mutant. This finding demonstrates that the levels of the CAT1 and CAT3 proteins are below the detection level in leaves (Figure 2b, upper blot). Single immune‐reactive bands co‐migrating with the wild type CAT2 tetramer were observed in the CAT2^WSQV^ and CAT2^PSI^ leaves. However, the CAT2 reactive band migrated more slowly in the CAT2^ARL^ samples than the other lines. We interpret this observation in terms of an increase in net charge of +4 in the tetramer (arrow, Figure 2a, upper blot). The upper band observed in Figure 2 is an unrelated cross‐reacting protein (possibly RUBISCO). The identity of the lower molecular weight bands observed in Figure 2 was confirmed as CAT by activity staining (Figure 2b, lower panel).

### 3.3 CAT2 C‐terminal variants restore cellular redox status

**Figure 3:**
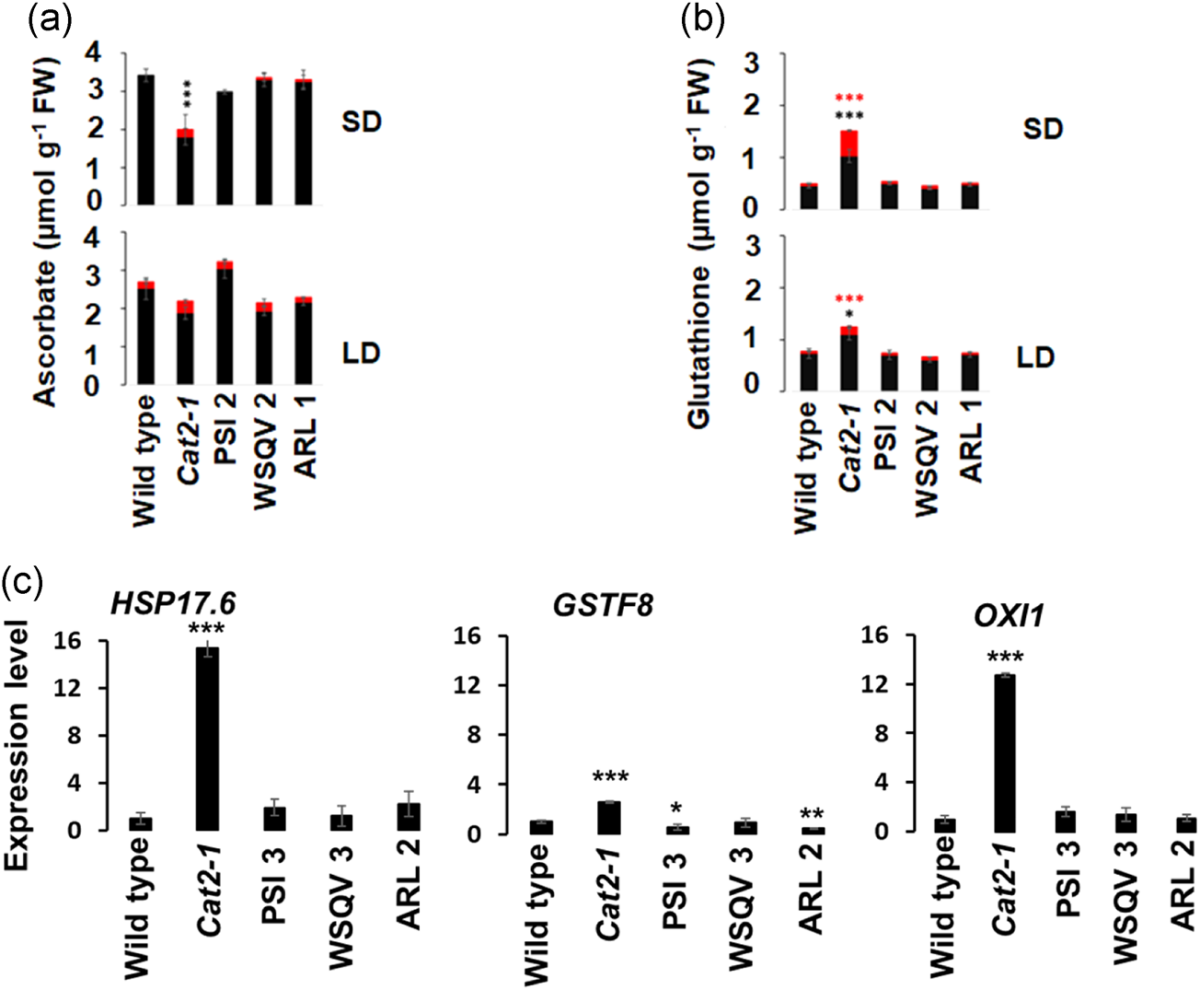
Redox balance and oxidative signalling are restored in complemented lines. Wild type, *cat2‐1* mutant, and transgenic lines were grown under short (SD; 8hL 16hD) for 4 weeks and then transferred into long‐day (LD; 16h L8hD) conditions for 1 week and measurements made of reduced and oxidised ascorbate (a) and glutathione (b). In each case, black bars represent the reduced form and red bars the oxidised form. ***p < 0.001 by Student's *t*‐test compared with the wild type (Content of the reduced form). GSSG and GSH ratio was also calculated (SD; 10:1, 2:1, 12:1, 7:1, 15:1 for wild type, *cat2‐1*, CAT2^PSI2^ CAT2^WSQV2^ and CAT2^ARL1^, respectively); (LD; 17:1, 7:1, 18:1, 11:1, 21:1 for wild type, *cat2‐1*, CAT2^PSI2^ CAT2^WSQV2^ and CAT2^ARL1^, respectively). Data are means of three independent extracts of three different plants. Error bars represent the SE. Black asterisks (content of GSH) or red (content of GSSG). (c) RT‐qPCR analysis of oxidative marker transcripts from plants grown under SD conditions for 4 weeks. The mean value of three replicates was normalised using *ACTIN 2* as the internal control. The expression level in the wild type was assigned a value of 1. (*p < 0.05) and (***p < 0.001) by Student's *t‐*test compared to the wild type [Color figure can be viewed at wileyonlinelibrary.com]

CAT2 deficiency results in H_2_O_2_ accumulation that perturbs cellular redox status, resulting in changes in the levels and oxidation state of nonenzymatic antioxidants, such as ascorbate and glutathione (Noctor & Foyer, 1998). The *cat2‐1* mutants had lower levels of ascorbate when grown under short‐day conditions, together with an increase in the proportion of oxidised ascorbate compared to the wild type plants (Figure 3a, SD). This finding is consistent with previous reports (Mhamdi et al., 2010; Queval et al., 2007). The level of ascorbate and increased ratios of reduced to oxidised ascorbate were restored to wild type levels in all the transgenic lines under short‐day growth conditions. The ascorbate levels were similar in all lines when plants were transferred to long‐day growth conditions for 1 week (Figure 3a, LD). However, the ascorbate pool was more oxidised in all lines under long‐day growth conditions relative to short days.

The level of total glutathione (reduced glutathione [GSH] plus glutathione disulphide [GSSG]) was dramatically increased in the *cat2‐1* leaves compared with the wild type under both short‐day and long‐day growth conditions (Figure 3b, SD, LD). These findings are consistent with previous studies (Mhamdi et al., 2010; Queval et al., 2007). The level of total glutathione and the GSH/GSSG ratios were similar in the transgenic lines and wildtype under both short‐ and long‐day conditions.

The levels of marker transcripts (*HSP17.6*, *GSTF8* and *OXI1*) for enhanced H_2_O_2_ accumulation were also examined. Accumulation of the selected transcripts has been shown to occur under oxidative stress (Noctor et al., 2016; Queval et al., 2007; Vanderauwera et al., 2005). The levels of the *HSP17.6*, *OXI1* and *GSTF8* transcripts were higher in the leaves of the *cat2‐1* mutants than the wild type plants that had been grown under short‐day conditions for 4 weeks (Figure 3c). However, the levels of marker transcripts were similar in the transgenic lines and the wild type under these conditions.

### 3.4 CAT2 variants target to peroxisomes

All the CAT2 variants had activity and could completely or partially (in the case of ARL) complement the various *cat2‐1* mutant phenotypes. However, a key question is whether CAT2 was localised within peroxisomes. Four‐week‐old rosettes of wild type, *cat2‐1* and one of each of the transgenic lines were fractionated into a clarified homogenate (S1) a cytosol fraction (S2) and an organelle pellet (P2). Protein and catalase activity were measured in each fraction along with hydroxypyruvate reductase (HPR) as a peroxisomal marker and expressed as activity/g fresh weight (Table 1). Peroxisomes are fragile and only 13%–42% of HPR activity was recovered in the organelle pellet. Between 41% and 78% of catalase activity was in the organelle pellet and that remaining soluble was most likely lost from organelles broken during the fractionation procedure. Calculation of the specific activity of catalase in the organelle pellet showed this was restored to wild type levels in CAT2^PSI^ and CAT2^WQSV^ but was lower in CAT2^ARL^ (Figure S8). Interestingly the activity of HPR was much lower in the *cat2‐1* mutant compared to the wild type or complemented lines (Table 1). The reason for this is not known but catalase has been reported to have a protective effect on peroxisomal isocitrate lyase (Yanik & Donaldson, 2005)

**Table 1:** Catalase and hydroxypyruvate reductase activities in cell fractions of Arabidopsis rosette leaves

Fraction	WT	*cat2‐1*	CAT2^PSI^	CAT2^WSQV^	CAT2^ARL^
Catalase S1 μmol/min/gfwt	438.0	97.0	320.0	322.5	286.5
Catalase S2 μmol/min/gfwt	257.5	45.5	193.0	196.5	218.0
Catalase P2 μmol/min/gfwt	177.5	40.5	252.5	209.5	117.0
% Catalase recovery	99	89	139	126	117
% Catalase in P2	41	42	78	65	41
HPR S1 μmol/min/gfwt	1899.5	686.0	1785.5	2121.5	2332.5
HPR S2 μmol/min/gfwt	1466.0	518.5	1143.5	1525.5	1690.0
HPR P2 μmol/min/gfwt	424.0	181.5	753.0	265.0	616.0
% HPR recovery	100	102	106	84	99
% HPR in P2	22	26	42	13	26

**Figure 4:**
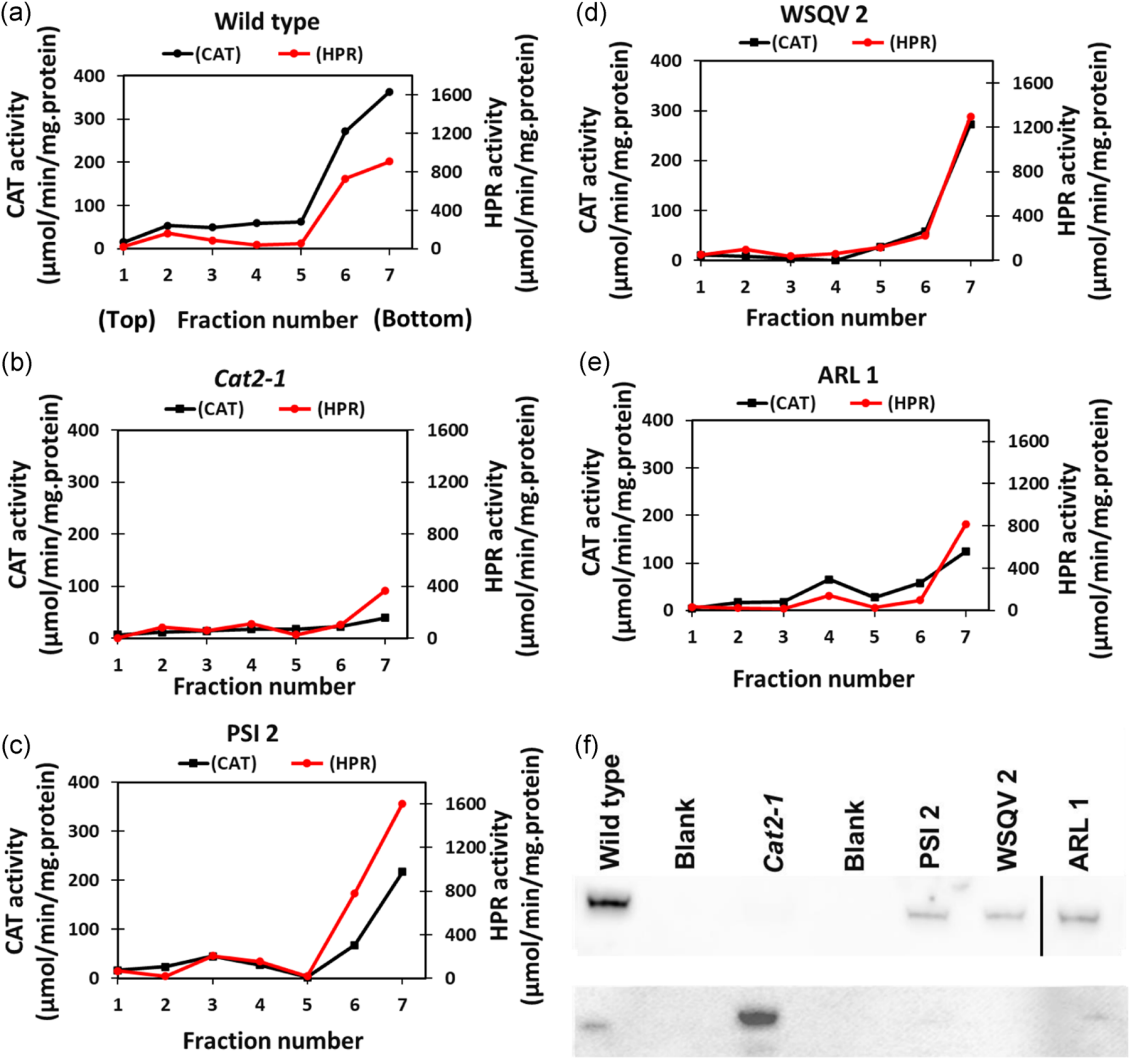
Cell fractionation reveals all catalase forms are targeted to peroxisomes. Subcellular fractionation of 4‐week‐old *Arabidopsis thaliana* leaves of wild type, *cat2‐1* mutant, and transgenic lines (a–f). A crude peroxisome pellet of the indicated lines was separated on a sucrose gradient and hydroxypyruvate reductase (HPR, red) and catalase (black) specific activities were determined in each fraction. The fractions corresponding to the peroxisomes were separated by SDS‐PAGE and immunoblotted with anti‐CAT2 (f, upper panel) and anti‐Atpβ antibodies (f, lower panel). The vertical black line in panel (f) upper panel indicates where a lane was moved to produce the final figure. The original image can be seen in Figure S11C [Color figure can be viewed at wileyonlinelibrary.com]

As the P2 fraction will contain other organelles, fractions of highly purified leaf peroxisomes were isolated using a combination of Percoll and sucrose density gradients (Reumann et al., 2007). In contrast to chloroplasts that were retained close to the top of the Percoll gradient, peroxisomes passed through the Percoll layer and were recovered at the bottom, as determined by analysis of the activity of the peroxisomal marker enzyme hydroxypyruvate reductase (HPR; data not shown). The isolated peroxisomes were then applied to a discontinuous sucrose density gradient. Chlorophyll was not detected in any of the fractions from the sucrose density gradient. The distribution of CAT activity was the same as HPR, providing strong evidence for the peroxisomal location of CAT (Figure 4). While the CAT activity of peroxisomes was very low in the *cat2‐1* mutants, the peroxisomes from the CAT2^PSI^ and CAT2^WSQV^ lines had similar activities to the wild type. In contrast, CAT activity was lower in the CAT2^ARL^ line peroxisomes, consistent with the data presented in Figure 2a.

The peroxisomal fraction was analysed using SDSPAGE and immunoblotting with antibodies against the β subunit of mitochondrial F1 ATPase, as well as CAT2. CAT2 was detected in the peroxisomal fractions from all lines except the *cat2‐1* mutant (Figure 4f, top panel). The wild type peroxisomal fraction showed a minor contamination by mitochondria, as indicated by the level of cross‐reaction with the ATPβ antibody (Figure 4f, bottom panel). Very minor contamination of the peroxisomes with mitochondria was observed in fractions from the transgenic lines. These data support the conclusion that CAT2 is localised in the peroxisomes of the transgenic lines. The *cat2‐1* peroxisome fraction showed a higher degree of contamination than other lines, which may reflect the altered density of peroxisomes lacking their most abundant protein.

### 3.5 Dynamic localisation of catalase using self‐assembling split GFP

**Figure 5:**
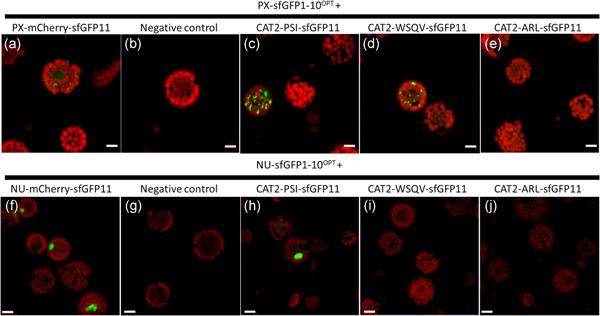
C terminal tagged wild type CAT2 and short‐form target to peroxisomes, wild type can also target to nucleus. CAT2 variants tagged with sfGFP11 at the C terminus transfected into protoplasts of transgenic Arabidopsis expressing sfGFP1‐10^OPT^ targeted to peroxisome (top) and nucleus (bottom). (b,f) Positive control: PX‐mCherry‐11and NU‐mCherry‐11 for peroxisome and nucleus, respectively. (b,g) Negative control (no plasmid). (c,h) CAT2^PSI^‐sfGFP11. (d,i) CAT2^WSQV^‐sfGFP11. (f,j) CAT2^ARL^‐sfGFP11. Scale bars = 10 µm (top) and 20 µm (bottom). Representative images reflecting the results of 3 (top) and 2 (bottom) independent experiments are shown. Protoplasts were incubated for 27–29 h in light and scanned using confocal laser scanning microscopy [Color figure can be viewed at wileyonlinelibrary.com]

We investigated the subcellular localisation of *At*CAT2 further using the self‐assembling split sfGFP^OPT^ system (Park et al., 2017), together with confocal laser scanning microscopy. For this analysis, CAT2 variants fused to sfGFP11 (at the C‐terminal end) were expressed in Arabidopsis protoplasts expressing sfGFP1‐10 β‐strand (sfGFP1‐10^OPT^) targeted to peroxisomes or nucleus (Figure 5). Positive controls (Park et al., 2017) were used where the appropriate organelle‐targeted mCherry‐sfGFP11 was expressed in protoplasts and labelled peroxisomes (Figure 5a) and nucleus (Figure 5f), respectively. No GFP signal was detected in the negative control (minus a GFP11construct) and no overlap with chlorophyll florescence was observed (Figure 5b,g). Expression of CAT2^PSI^‐sfGFP11 and CAT2^WSQV^‐sfGFP11 in protoplasts isolated from peroxisome‐targeted sfGFP1‐10^OPT^ (PX‐sfGFP1‐10^OPT^) reconstituted sfGFP fluorescence signal in peroxisomes (Figure 5c,d). This finding suggests that CAT is targeted to peroxisomes in these lines. In contrast, no signal was detected with CAT2^ARL^ (Figure 5e) with the exception of just two protoplasts in one experiment where weak fluorescence was detected in unknown membranous structures (Figure S9). Protoplasts were also isolated from the transgenic lines expressing sfGFP1‐10^OPT^ targeted to the nuclei (Nu‐sfGFP1‐10^OPT^). A reconstituted sfGFP signal was observed in the nuclei of the protoplasts transfected with CAT2^PSI^‐sfGFP11 construct Figure 5h). In contrast, no signal was detectable in the protoplasts transfected with CAT2^WSQV^ or ^ARL^‐sfGFP11 variants (Figure 5i,j). These results were highly reproducible and were observed in multiple protoplasts in independent experiments (Table S3).

**Figure 6:**
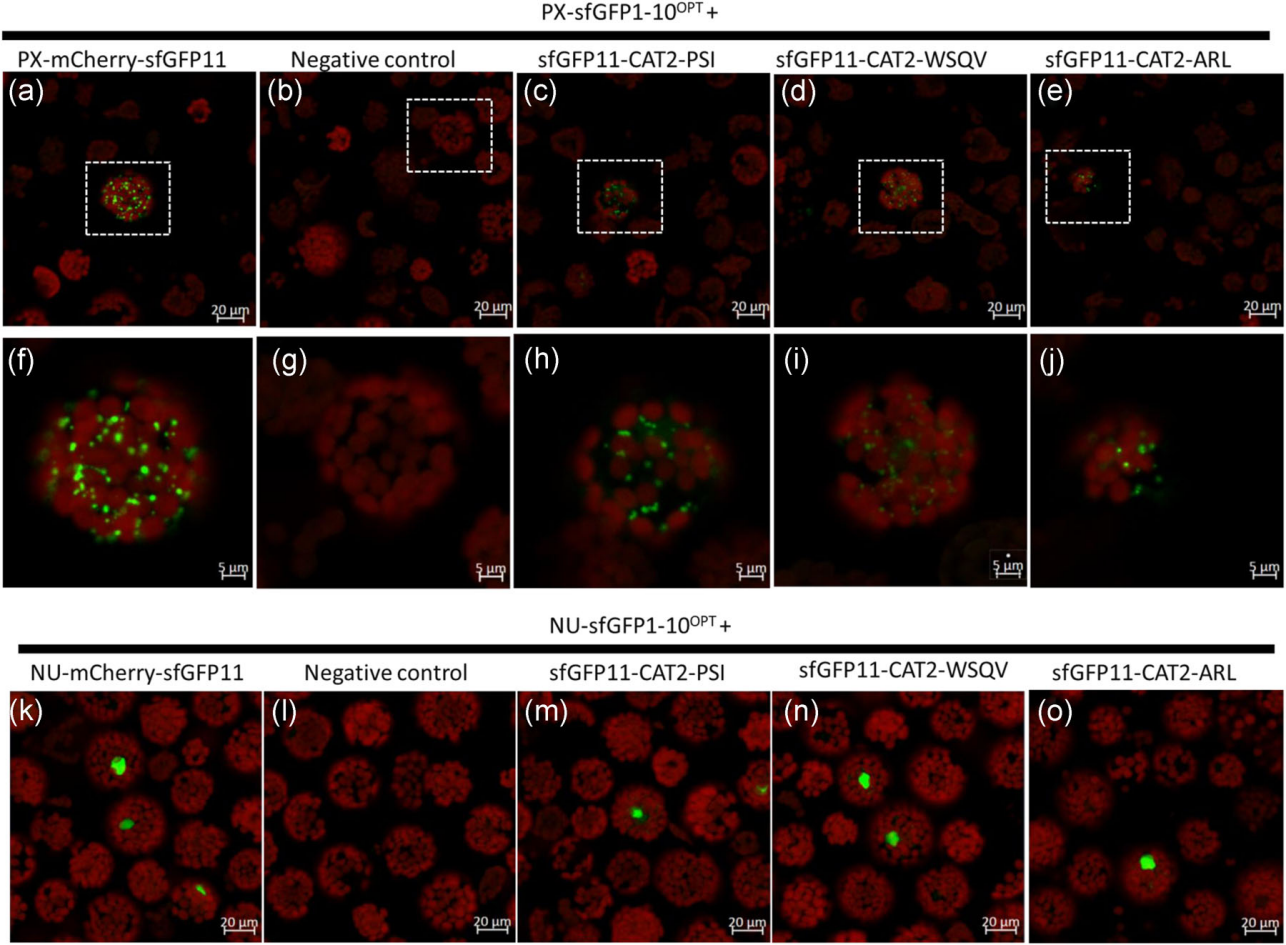
N terminal tagged CAT2 variants target to nucleus and peroxisomes. CAT2 variants tagged with sfGFP11 at the N terminus transfected into protoplasts of transgenic Arabidopsis expressing sfGFP1‐10^OPT^ targeted to peroxisome (a–j) and nucleus (k–o). The middle row (f–j) shows a magnified image of the protoplast indicated by a white dotted box in the panel above. Positive control: PX‐mCherry‐11 (a,f) and NU‐mCherry‐11 (k) for peroxisome and nucleus, respectively. (b,g,l) Negative control (no plasmid). (c,h,m) sfGFP11‐CAT2^PSI^. (d,l,n) sfGFP11‐CAT2^WSQV^. (e,j,o) sfGFP11‐CAT2^ARL^. Scale bars = 20 µm top and bottom 5 μm middle. Representative images reflecting the results of 1 (top) and 2 (bottom) independent experiments are shown. Protoplasts were incubated for 27–29 h in light and scanned using confocal laser scanning microscopy [Color figure can be viewed at wileyonlinelibrary.com]

When the GFP11 was placed at the N terminus of the constructs; sfGFP11‐CAT2^PSI^, sfGFP11‐CAT2^WSQV^ and sfGFP11‐CAT2^ARL^, a different result was obtained (Figure 6). Peroxisomal targeting was observed when all three constructs were expressed in protoplasts derived from PX‐sfGFP1‐10^OPT^ lines (Figure 6c–e and h–j magnified image). Nuclear targeting was observed for all three variants when the N terminal tagged constructs were expressed in protoplasts derived from Nu‐sfGFP1‐10^OPT^ plants (Figure 6m–o, bottom panel).

It was previously shown (Park et al., 2017) that mCherry sfGFP11 targeted to the nucleus or peroxisome could reconstitute fluorescence in the appropriate organelle when co‐expressed with cytosolic sfGFP1‐10^OPT^, presumably because the sfGFP11 and sfGFP1‐10 can assemble in the cytosol and be subsequently imported since these organelles can import folded proteins. To determine whether the reconstitution of fluorescence in the nucleus by the sfGFP11CAT2 proteins could be due to assembly with Nu‐sfGFP1‐10^OPT^ in the cytosol followed by nuclear import, both C and N terminal tagged CAT constructs were co‐expressed with CYTO‐sfGFP1‐10^OPT^(Figure S10). Surprisingly none of these constructs could reconstitute fluorescence in any compartment, suggesting that unlike cytosolic mCherryGFP11 the CAT constructs could not assemble with sfGFP1‐10^OPT^ in the cytosol and that the CAT constructs are imported into the nucleus where they assemble with Nu‐sfGFP1‐10^OPT^.

## 4 DISCUSSION

There is little consensus regarding the importance of sequences within the conserved C terminal region of catalase in targeting to peroxisomes (Fujikawa et al., 2019; Kamigaki et al., 2003; Mullen et al., 1997). Previous studies have used different approaches that rendered interpretation of the findings difficult. To resolve this problem, we addressed the question using a system that is as physiologically relevant as possible, by expressing untagged *At*CAT2 variants under the native CAT2 promoter in the *cat2‐1* mutant. This allowed testing not only of catalase targeting but crucially of functionality. Our data clearly demonstrate that the last 18 amino acids of CAT2 are not required for activity, peroxisome location or ability to complement any of the *cat2‐1* mutant phenotypes under any of the growth conditions tested.

Intriguingly the mutation of the terminal 3 amino acids of wild type CAT2 –PSI‐COOH to a PTS1 consensus sequence –ARL‐COOH led to only a partial complementation of some phenotypes. Whilst the CAT2^ARL^ variant was assembled into tetramers and targeted to peroxisomes, its activity was reduced. This is consistent with the finding that exchanging the C‐terminal tripeptide SKI of *Hansenula polymorpha* catalase for the consensus PTS1 sequence SKL resulted in reduced catalase activity in cell lysates and the formation of catalase aggregates in peroxisomes, leading the authors to propose that a lower affinity of the catalase PTS1 for PEX5 resulted in slower import, allowing time for catalase maturation in the cytosol (Williams et al., 2012). The folding and maturation pathway of catalase remains unclear. To produce a mature catalase protein, the subunits have to fold, bind haem and tetramerise. In *A. thaliana*, the NCA1 protein interacts with catalase in the cytosol to promote the formation of active enzyme; *nca1* mutants lack catalase activity and are hypersensitive to multiple stresses (Li et al., 2015) and immunity mediated autophagy (Hackenberg et al., 2013).

The *H. polymorpha* catalase C terminal sequence SKI binds PEX5 with 8‐fold reduced affinity compared to an equivalent peptide with the C terminal sequence SKL (Williams et al., 2012). In contrast, a peptide corresponding to the C terminus of *At*CAT2 showed no binding to either full‐length PEX5 or the C terminal TPR domain (Skoulding, Baker Warriner unpublished), while a much larger portion of pumpkin CAT1 comprising the C terminal 150 amino acids interacted with the N terminal part of PEX5 (Oshima et al., 2008). *S. cerevisae* peroxisomal catalase Cta1p is also independent of the PEX5 TPR domain for import (Rymer et al., 2018), and mammalian catalase interacts with the N terminal part of PEX5 and this interaction blocks catalase tetramerisation (Freitas et al., 2011). Collectively, these data point to a different mode of interaction of catalase with PEX5 which depends upon attaining a properly folded structure. This could be because noncontiguous amino acids in the primary sequence come together in the folded structure to form a targeting determinant as has been recently shown for *S. cerevisae* acyl CoA oxidase (Kempiński et al., 2020). The requirement for a properly folded catalase for import competence is also suggested by the finding that mutations in the haem binding site of Arabidopsis CAT2 interfere with peroxisomal localisation (Fujikawa et al., 2019). In principle a competition could exist between catalase binding to PEX5 for import and NCA1 binding for proper maturation. Intriguingly both contain TPR domains which are important for interacting with their substrates (Gatto et al., 2003; Li et al., 2015). Thus, providing a consensus PTS1 sequence ARL might commit CAT2 apoproteins to the peroxisome import pathway before they have time to fold and bind haem. By expressing untagged catalase variants under the control of the CAT2 promoter in the Arabidopsis *cat2‐1* background as stable transformants, all folding and maturation factors and their substrates are present at natural levels and issues of overexpression, interspecies incompatibility or tag impairment of folding are therefore avoided. Thus, it can be confidently concluded that the C‐terminal 18 amino acids of CAT2 are not required for activity or peroxisome targeting.

The above discussion leads to the question of why the C‐terminal 18 amino acids of CAT2 is so highly conserved, if the sequence is apparently dispensable. Cell fractionation is not appropriate for studying dynamic changes in protein localisation so the superfolder split GFP system was employed. This system was developed to study translocation of type III secretion system substrates which are folded and whose translocation is often prevented when fused to large fluorescent proteins (Park et al., 2017). The 11th β strand of GFP (GFP11) is only 16 amino acids, not much larger than an epitope tag, and should be minimally invasive when fused to a target protein. When GFP11 was fused to the C terminus of either CAT2^PSI^ or CAT2^WSQV^ and expressed in protoplasts derived from plants with GFP1‐10 targeted to peroxisomes, these constructs targeted to peroxisomes demonstrating that a free C terminus was not required. Conversely, CAT2^ARL^ was not targeted to peroxisomes when GFP11 was appended to the C terminus, as might be expected if the protein now depends upon canonical PTS1‐PEX5 interaction which requires a free carboxylate on the C terminal leucine residue. However, it appears not to be able to access the pathway used by CAT2^PSI^ and CAT2^WSQV^. Finally, the 3 catalase variants were co‐expressed with nuclear‐targeted GFP1‐10. CAT2^PSI^ was efficiently targeted to the nucleus whether the sfGFP11 was at the N or C terminus but CAT2^ARL^ and CAT2^WQSV^ could only target to the nucleus when the sfGFP11 was at the N terminus, suggesting that alteration of the C terminus in combination with a C terminal tag interferes with the recognition by some unknown factor that promotes nuclear targeting (Figures 5 and 6).

Catalases can be relocated to the nucleus through the interaction with effector proteins of *Phytopthora sojae* to manipulate PCD (M. Zhang et al., 2015) and the cucumber mosaic virus (CMV) protein 2b leading to viral‐induced necrosis (Inaba et al., 2011; Murota et al., 2017). An effector protein from the plant growth promoting rhizobacterium *Saccharothryx yanglingensis* interacts with catalase in the nucleus and stimulates plant immunity (Y. Zhang et al., 2018). Thus, redirection of catalase to the nucleus appears to be a strategy employed by microorganisms to modulate the outcomes of their interactions with plants. Our results demonstrate that catalase has the capacity to be transported to the nucleus independent of any exogenous effector proteins. This is a specific response and not a consequence of cytosolic assembly with nuclear targeted GFP1 −10 followed by import since CAT2‐WSQVsfGFP11 and CAT2‐ARLsfGFP11 were not nuclear. Furthermore, CAT2 constructs were not able to assemble with cytosolic GFP1‐10 which argues for their autonomous import and subsequent assembly with nuclear‐targeted GFP1‐10. This finding may explain the reported interaction of catalase with nucleoredoxin 1 (NRX1; Kneeshaw et al., 2017) and suggests that catalase dynamics could be a component of innate plant responses and that the highly conserved C terminal region plays an important role in this behaviour.

## CONFLICT OF INTERESTS

The authors declare that there are no conflict of interests.

## AUTHOR CONTRIBUTIONS

**Yousef Al‐Hajaya:** Designed and performed experiments, analysed data, wrote the manuscript. **Barbara Karpinska:** Designed and performed experiments, analysed data. **Christine H. Foyer:** Designed and analysed experiments, wrote the manuscript. **Alison Baker:** Designed and analysed experiments, wrote the manuscript.

## Supporting information

Supporting information.

## Data Availability

The data that supports the findings of this study are available in the supporting information of this article.
