# Supplementary material for: Nuclear and peroxisomal targeting of catalase
Source: Plant Cell Environ. 2022 Jan 27;45(4):1096–108. doi: 10.1111/pce.14262 (PMC9305541; doi:10.1111/pce.14262)
Supplement: Supplementary file 1 — Supporting information. [file PCE-45-1096-s001.docx]

**Supporting Information**

**Materials and Methods**

**Root measurements**. Wild type, *cat2-1* mutant and transgenic lines were sown on ½ MS plates (supplemented with Hygromycin for the transgenic lines) and kept in the cold room for two days. After cold treatment, plates were moved into the light for 6hrs, then kept for two days in the dark. Plates were then moved into 16hr photoperiod. Ten days after germination, plates were photographed using a Nikon D5100 digital camera. Image J 1.48 V programme was used to analyse the images.

**Supplementary Figures**

**Figure S1 Plasmid maps of CAT2 variants**

**
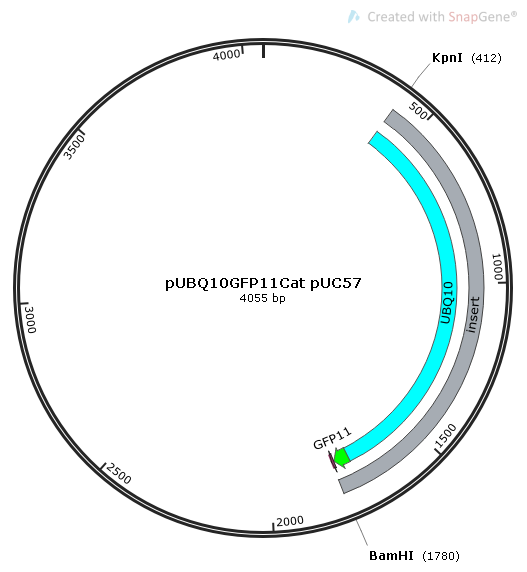
Figure S2 Plasmid map and sequence of pUBQ10GFP11Cat-pUC57**

>2_pUBQ10GFP11Cat_pUC57

TCGCGCGTTTCGGTGATGACGGTGAAAACCTCTGACACATGCAGCTCCCGGAGACGGTCACAGCTTGTCTGTAAGCGGATGCCGGGAGCAGACAAGCCCGTCAGGGCGCGTCAGCGGGTGTTGGCGGGTGTCGGGGCTGGCTTAACTATGCGGCATCAGAGCAGATTGTACTGAGAGTGCACCATATGCGGTGTGAAATACCGCACAGATGCGTAAGGAGAAAATACCGCATCAGGCGCCATTCGCCATTCAGGCTGCGCAACTGTTGGGAAGGGCGATCGGTGCGGGCCTCTTCGCTATTACGCCAGCTGGCGAAAGGGGGATGTGCTGCAAGGCGATTAAGTTGGGTAACGCCAGGGTTTTCCCAGTCACGACGTTGTAAAACGACGGCCAGTGAATTCGAGCTC**GGTACC**CTCGAGGATCAGGATATTCTTGTTTAAGATGTTGAACTCTATGGAGGTTTGTATGAACTGATGATCTAGGACCGGATAAGTTCCCTTCTTCATAGCGAACTTATTCAAAGAATGTTTTGTGTATCATTCTTGTTACATTGTTATTAATGAAAAAATATTATTGGTCATTGGACTGAACACGAGTGTTAAATATGGACCAGGCCCCAAATAAGATCCATTGATATATGAATTAAATAACAAGAATAAATCGAGTCACCAAACCACTTGCCTTTTTTAACGAGACTTGTTCACCAACTTGATACAAAAGTCATTATCCTATGCAAATCAATAATCATACAAAAATATCCAATAACACTAAAAAATTAAAAGAAATGGATAATTTCACAATATGTTATACGATAAAGAAGTTACTTTTCCAAGAAATTCACTGATTTTATAAGCCCACTTGCATTAGATAAATGGCAAAAAAAAACAAAAAGGAAAAGAAATAAAGCACGAAGAATTCTAGAAAATACGAAATACGCTTCAATGCAGTGGGACCCACGGTTCAATTATTGCCAATTTTCAGCTCCACCGTATATTTAAAAAATAAAACGATAATGCTAAAAAAATATAAATCGTAACGATCGTTAAATCTCAACGGCTGGATCTTATGACGACCGTTAGAAATTGTGGTTGTCGACGAGTCAGTAATAAACGGCGTCAAAGTGGTTGCAGCCGGCACACACGAGTCGTGTTTATCAACTCAAAGCACAAATACTTTTCCTCAACCTAAAAATAAGGCAATTAGCCAAAAACAACTTTGCGTGTAAACAACGCTCAATACACGTGTCATTTTATTATTAGCTATTGCTTCACCGCCTTAGCTTTCTCGTGACCTAGTCGTCCTCGTCTTTTCTTCTTCTTCTTCTATAAAACAATACCCAAAGAGCTCTTCTTCTTCACAATTCAGATTTCAATTTCTCAAAATCTTAAAAACTTTCTCTCAATTCTCTCTACCGTGATCAAGGTAAATTTCTGTGTTCCTTATTCTCTCAAAATCTTCGATTTTGTTTTCGTTCGATCCCAATTTCGTATATGTTCTTTGGTTTAGATTCTGTTAATCTTAGATCGAAGACGATTTTCTGGGTTTGATCGTTAGATATCATCTTAATTCTCGATTAGGGTTTCATAAATATCATCCGATTTGTTCAAATAATTTGAGTTTTGTCGAATAATTACTCTTCGATTTGTGATTTCTATCTAGATCTGGTGTTAGTTTCTAGTTTGTGCGATCGAATTTGTCGATTAATCTGAGTTTTTCTGATTAACAGATGCGGGACCACATGGTGCTGCACGAGTACGTGAACGCCGCCGGCATCACAAT**GGATCC**CGGGCCCGTCGACTGCAGAGGCCTGCATGCAAGCTTGGCGTAATCATGGTCATAGCTGTTTCCTGTGTGAAATTGTTATCCGCTCACAATTCCACACAACATACGAGCCGGAAGCATAAAGTGTAAAGCCTGGGGTGCCTAATGAGTGAGCTAACTCACATTAATTGCGTTGCGCTCACTGCCCGCTTTCCAGTCGGGAAACCTGTCGTGCCAGCTGCATTAATGAATCGGCCAACGCGCGGGGAGAGGCGGTTTGCGTATTGGGCGCTCTTCCGCTTCCTCGCTCACTGACTCGCTGCGCTCGGTCGTTCGGCTGCGGCGAGCGGTATCAGCTCACTCAAAGGCGGTAATACGGTTATCCACAGAATCAGGGGATAACGCAGGAAAGAACATGTGAGCAAAAGGCCAGCAAAAGGCCAGGAACCGTAAAAAGGCCGCGTTGCTGGCGTTTTTCCATAGGCTCCGCCCCCCTGACGAGCATCACAAAAATCGACGCTCAAGTCAGAGGTGGCGAAACCCGACAGGACTATAAAGATACCAGGCGTTTCCCCCTGGAAGCTCCCTCGTGCGCTCTCCTGTTCCGACCCTGCCGCTTACCGGATACCTGTCCGCCTTTCTCCCTTCGGGAAGCGTGGCGCTTTCTCATAGCTCACGCTGTAGGTATCTCAGTTCGGTGTAGGTCGTTCGCTCCAAGCTGGGCTGTGTGCACGAACCCCCCGTTCAGCCCGACCGCTGCGCCTTATCCGGTAACTATCGTCTTGAGTCCAACCCGGTAAGACACGACTTATCGCCACTGGCAGCAGCCACTGGTAACAGGATTAGCAGAGCGAGGTATGTAGGCGGTGCTACAGAGTTCTTGAAGTGGTGGCCTAACTACGGCTACACTAGAAGAACAGTATTTGGTATCTGCGCTCTGCTGAAGCCAGTTACCTTCGGAAAAAGAGTTGGTAGCTCTTGATCCGGCAAACAAACCACCGCTGGTAGCGGTGGTTTTTTTGTTTGCAAGCAGCAGATTACGCGCAGAAAAAAAGGATCTCAAGAAGATCCTTTGATCTTTTCTACGGGGTCTGACGCTCAGTGGAACGAAAACTCACGTTAAGGGATTTTGGTCATGAGATTATCAAAAAGGATCTTCACCTAGATCCTTTTAAATTAAAAATGAAGTTTTAAATCAATCTAAAGTATATATGAGTAAACTTGGTCTGACAGTTACCAATGCTTAATCAGTGAGGCACCTATCTCAGCGATCTGTCTATTTCGTTCATCCATAGTTGCCTGACTCCCCGTCGTGTAGATAACTACGATACGGGAGGGCTTACCATCTGGCCCCAGTGCTGCAATGATACCGCGAGACCCACGCTCACCGGCTCCAGATTTATCAGCAATAAACCAGCCAGCCGGAAGGGCCGAGCGCAGAAGTGGTCCTGCAACTTTATCCGCCTCCATCCAGTCTATTAATTGTTGCCGGGAAGCTAGAGTAAGTAGTTCGCCAGTTAATAGTTTGCGCAACGTTGTTGCCATTGCTACAGGCATCGTGGTGTCACGCTCGTCGTTTGGTATGGCTTCATTCAGCTCCGGTTCCCAACGATCAAGGCGAGTTACATGATCCCCCATGTTGTGCAAAAAAGCGGTTAGCTCCTTCGGTCCTCCGATCGTTGTCAGAAGTAAGTTGGCCGCAGTGTTATCACTCATGGTTATGGCAGCACTGCATAATTCTCTTACTGTCATGCCATCCGTAAGATGCTTTTCTGTGACTGGTGAGTACTCAACCAAGTCATTCTGAGAATAGTGTATGCGGCGACCGAGTTGCTCTTGCCCGGCGTCAATACGGGATAATACCGCGCCACATAGCAGAACTTTAAAAGTGCTCATCATTGGAAAACGTTCTTCGGGGCGAAAACTCTCAAGGATCTTACCGCTGTTGAGATCCAGTTCGATGTAACCCACTCGTGCACCCAACTGATCTTCAGCATCTTTTACTTTCACCAGCGTTTCTGGGTGAGCAAAAACAGGAAGGCAAAATGCCGCAAAAAAGGGAATAAGGGCGACACGGAAATGTTGAATACTCATACTCTTCCTTTTTCAATATTATTGAAGCATTTATCAGGGTTATTGTCTCATGAGCGGATACATATTTGAATGTATTTAGAAAAATAAACAAATAGGGGTTCCGCGCACATTTCCCCGAAAAGTGCCACCTGACGTCTAAGAAACCATTATTATCATGACATTAACCTATAAAAATAGGCGTATCACGAGGCCCTTTCGTC

**
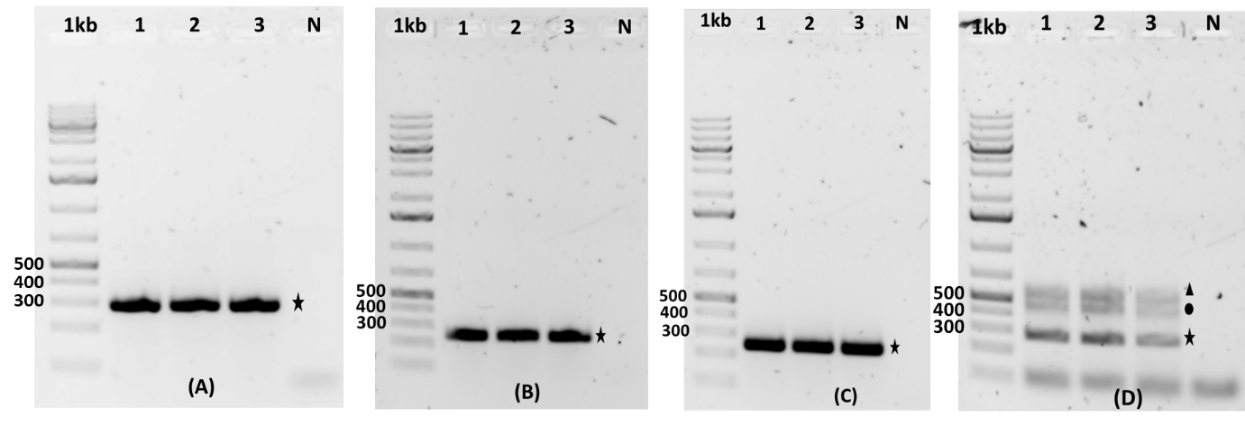
**

**Figure S3 *Arabidopsis thaliana* catalase 2 gene (*CAT2*) specific transcripts detection by RT-PCR.**

RT-PCR analysis to detect *CAT2* specific transcripts from polysomal RNA of wild type *Arabidopsis thaliana.* (A) PCR1 using (*CAT2*-F and *CAT2*-R) that allow amplification of both transcripts. Only one band (star, 293 bp) which corresponds to At4g35090.1 transcript was detected at 35 cycles. (B) and (C) are PCR2 at 25 and 30 cycles respectively, one band (star, 246 bp) which is also specific to At4g35090.1 transcript was detected using *CAT2-*F and *CAT2-1*R primer pair. (D) PCR3 with 40 cycles, At4g35090.2 specific transcript was detected (star, 276bp) using *CAT2*-F and *CAT2-2*R primer pair. 1kb, DNA molecular weight markers; positions of markers (base pair) are shown on the left. Lanes (1-3), three biological samples. H_2_O was included as a negative control of the PCR reaction (N). Additional bands are indicated by triangle and circle. Triangle (D) shows a band which is suggested to be another-un spliced variant. Unspecific band (confirmed by sequencing) is shown (circle, D).

**
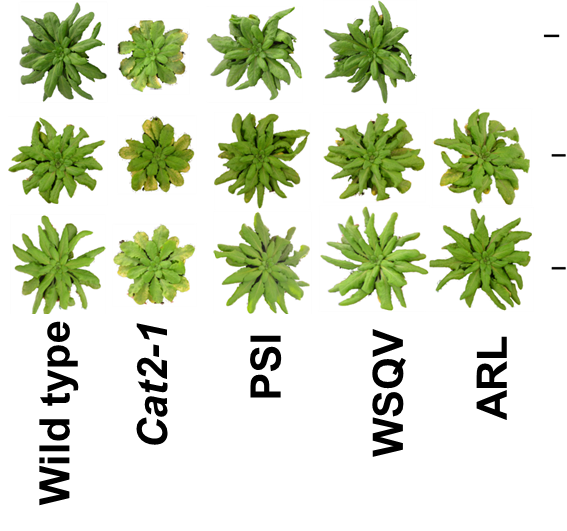
**

**Figure S4 Images of plants of the independent transgenic lines** Morphological analysis of wild type, *cat2-1* mutant and different independent lines transformed with CAT2 variants grown under long-day conditions (16h light/ 8h dark) for one week after growing for 4-5 weeks in short-day conditions. Scale bars=1 cm.

**
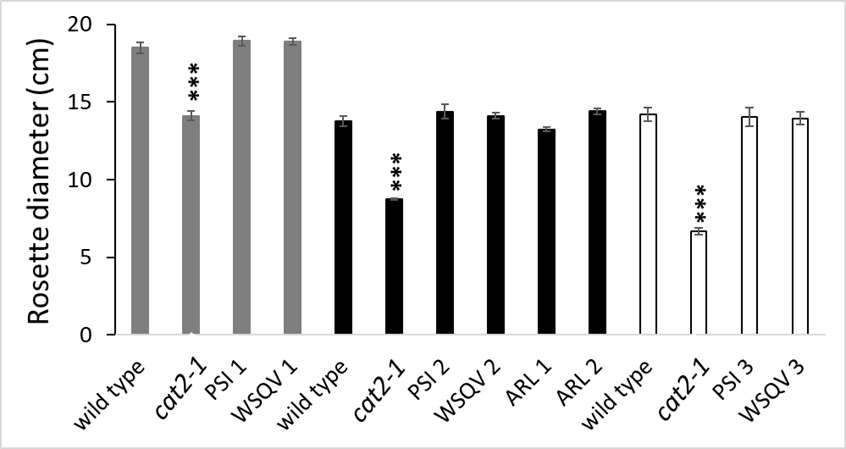
**

Figure S5. Rosette diameter (longest diameter) of wild type, *cat2-1,* and transgenic lines. Means were calculated form 24, 6 and 6 plants per genotype (grey, black and white, respectively, different coloured bars represent different experiments). Rosette diameter was determined using ImageJ 1.48V programme. *** P< 0.001, compared with wild type plants (Student’s t test).


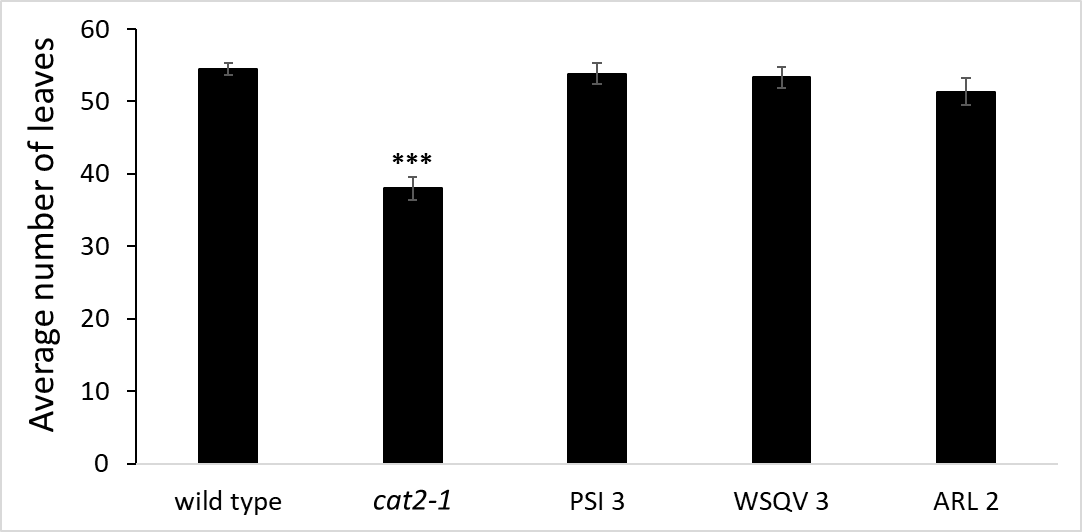


Figure S6. Number of leaves under long-day-conditions.

The total number of leaves was counted for 6 -week-old wild type, *cat2-1* mutant and CAT2 derivatives (3^rd^ independent line of PSI and WSQV; 2^nd^ independent line of ARL) grown under long-day conditions (16h light/8 h dark regime) for one week. Data are the mean values (n= 6) of each independent line. Asterisks indicate statistically significant differences compared with wild type by Student’s *t* test (*** P< 0.001).


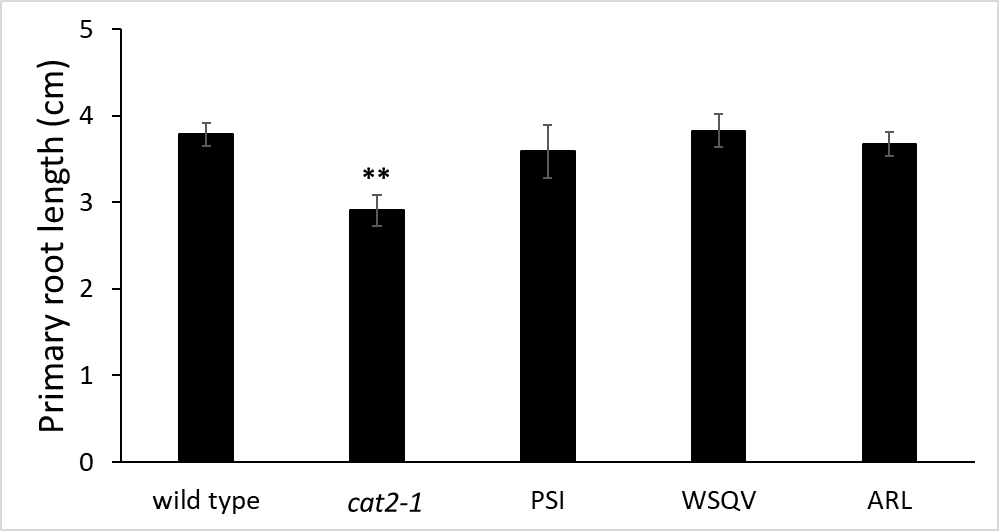


Figure S7. Primary root length (cm) in wild type, *cat2-1*, PSI, WSQV and ARL plants ten days after germination.

Data shown are means of 11 plants. ‘**’ significant difference from wild type at P < 0.01. Data are means of all the plants of that genotype combines (i.e. three independent lines of WSQV and PSI, as well as two independent lines of ARL were grown at the same time.


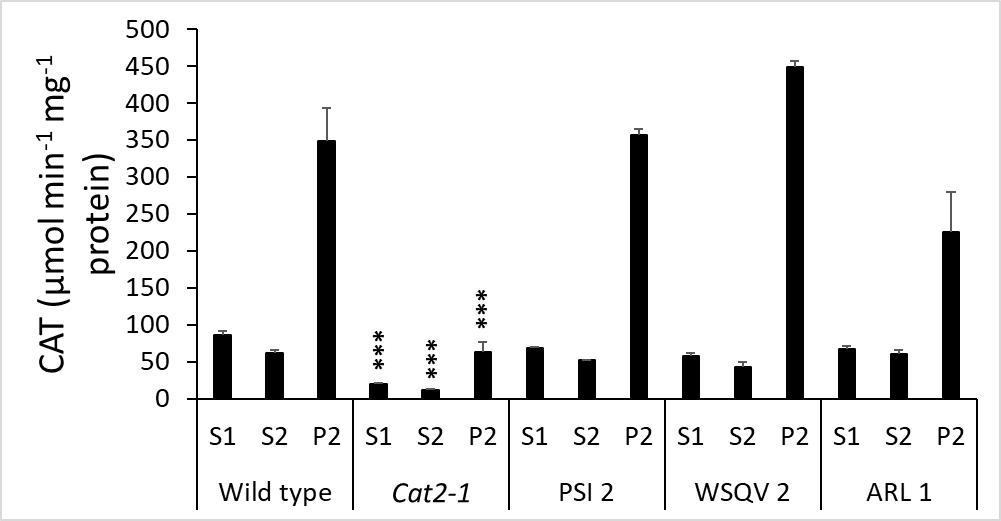


**Figure S8 Catalase activity is restored in the three fractions of transgenic lines compared with the wild type.**

Four weeks-old wild type, *cat2-1* mutant, PSI2, WSQV2 and ARL1 lines were homogenised and fractionated. Catalase specific activity (µmol min^-1^ mg^-1^ protein) was measured in homogenate (S1), cytosolic (S2) and organelle (P2) fraction of each line. Data are the means; all samples were analysed in triplicates; error bars are SE

**
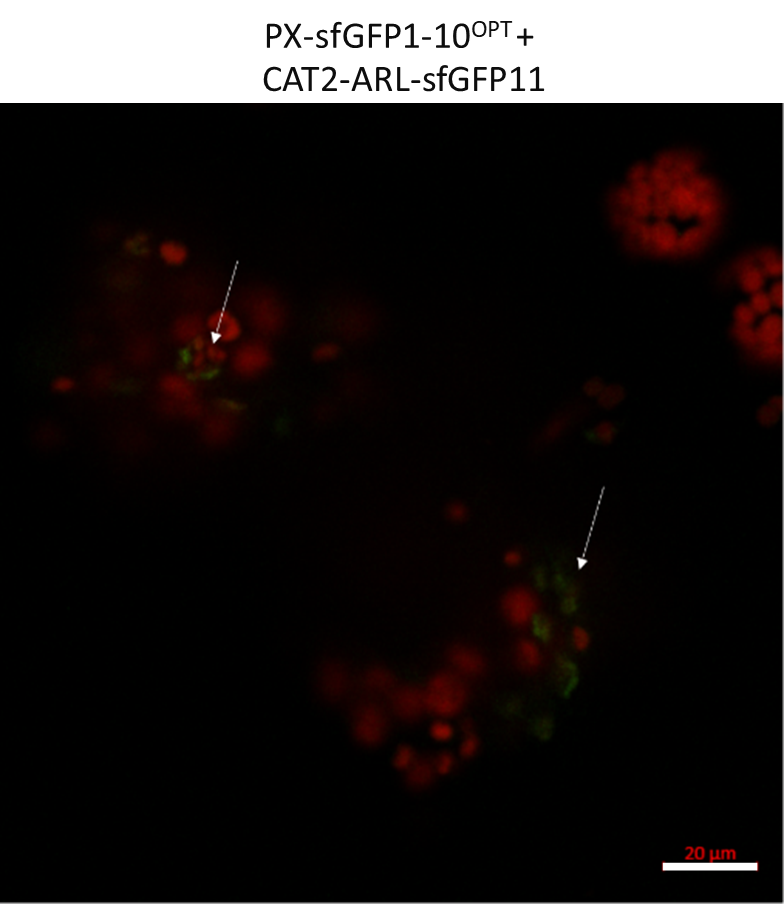
**

**Figure S9. CAT2ARL tagged with sfGFP11 is detectable in *A. thaliana* transgenic plants expressing peroxisome targeted sfGFP1-10^OPT^.** Reconstituted sfGFP signal (green, arrows) was detected in only two cells in a single experiment after 37hr incubation. The identity of the structures is unknown. Scale bar = 20 µm.

**Figure S10 Catalase constructs do not reconstitute fluorescence when co-expressed with CYTO sfGFP1-10.**

CAT2 variants tagged with sfGFP11 transfected into protoplasts of transgenic Arabidopsis expressing CYTO-sfGFP1-10^OPT^. Top; sfGFP11 was fused at the C terminus of the catalase constructs and bottom; sfGFP was fused at the N terminus of the catalase constructs. a positive control CYTO-mCherry-sfGFP11reconstututes fluorescence in the cytosol and nucleus. Negative control (b,f No plasmid). CAT2^PSI^ (c,g), CAT2^WSQV^ (d,h) CAT2^ARL^ (e,i). Scale bars = 10 µm (top) and 20 µm bottom).

**Figure S11 Original blots and gels that are presented in the paper**

**A and B figure 2**

**C and D figure 4**


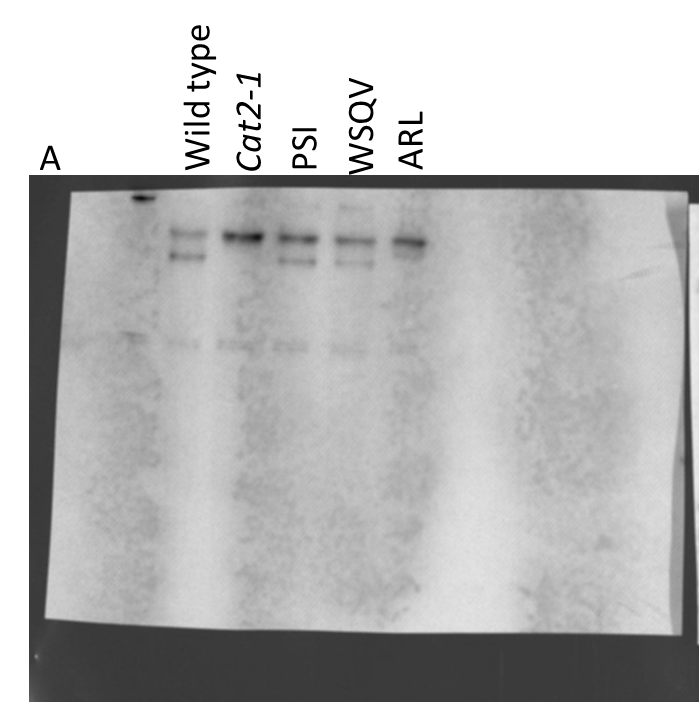


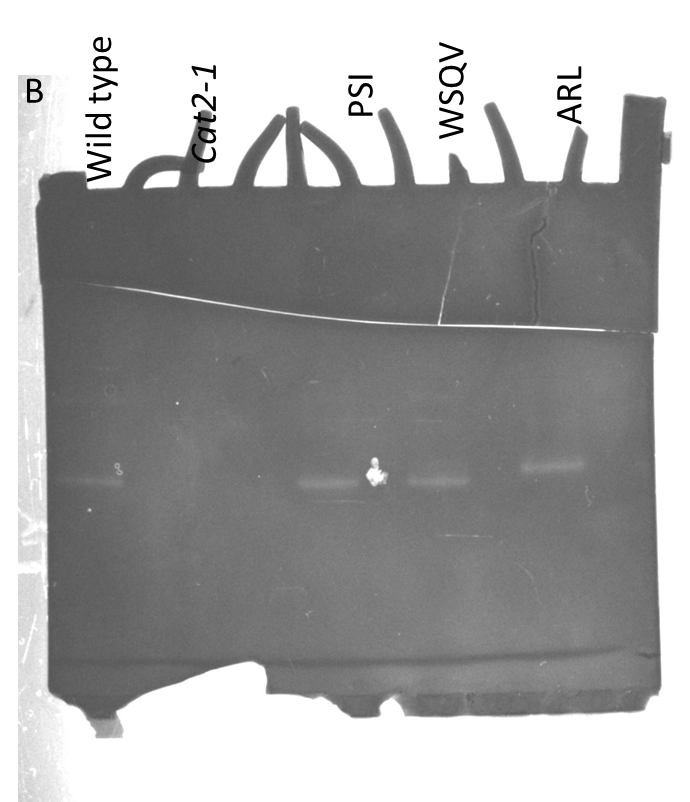


**
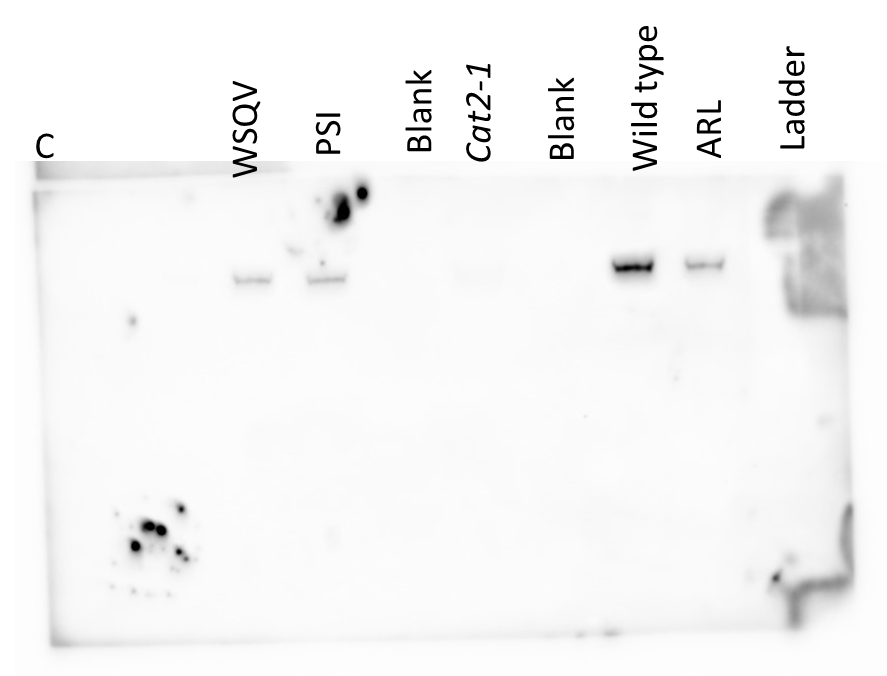
**

**
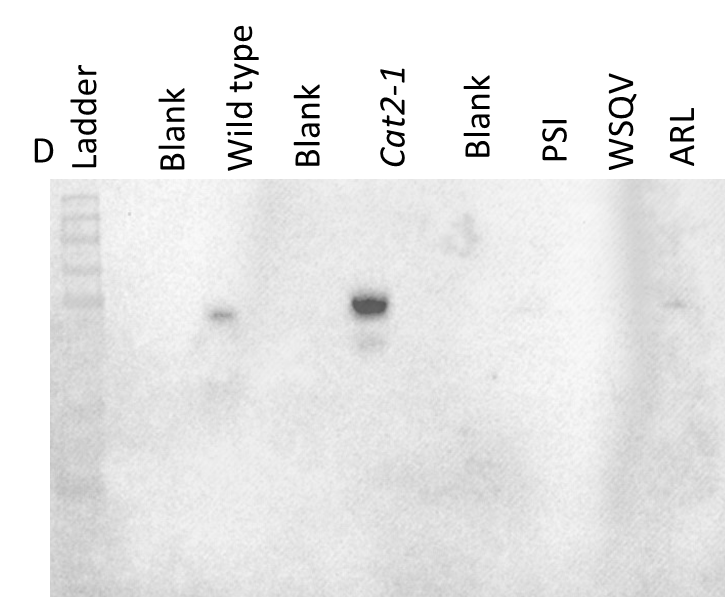
**

**Supplementary Tables**

**Table S1.** List of primers used in this study.

| N° | Names | primer sequence (5´ to 3´) | Purposes |
| --- | --- | --- | --- |
| 1 | ACT2up2 | TTCCCTCAGCACATTCCAGCAG | RT-qPCR |
| 2 | ACT2do | TTAACATTGCAAAGAGTTTCAAGG | RT-qPCR |
| 3 | qPCRgstF8up | CGAAGGTAAGCTCCAGAAAGTC | RT-qPCR |
| 4 | qPCRgstF8do | AGAGTCAAAGAGCACCTTGGAG | RT-qPCR |
| 5 | qPCRhsp17up | GGAGAAAGAAGATAAGAATGACACG | RT-qPCR |
| 6 | qPCRoxi1up | GTTGAGGAAATCAAGGGTCATG | RT-qPCR |
| 7 | qPCRoxi1do | TGGACGATATTCTCCACATCC | RT-qPCR |
| 8 | CTSPF2 | CTTACAACTCTCCCTTCTTCACCACCAACT | Sequencing |
| 9 | CTSPR2 | TGTTTGAACGATCGGGGAAATTCGA | Sequencing |
| 10 | CTSPR3 | ACCCGCCAATATATCCTGTCAAACAC | Sequencing |
| 11 | CAT2PSIF | TGGGTTGGTACCGATCCTTACAAGT ATCGTCCAGCTAGTTC | GFP11CAT2 construction |
| 12 | CAT2PSIR | GGTTCCACTAGTTTGATGCTTG  GTCTCACGTTCAGAC | GFP11CAT2 construction |
| 13 | CAT2WSQVR | GGTTCCACTAGTTTCACCTGAGACCA GTAAGAGATCCAG | GFP11CAT2 construction |
| 14 | CAT2ARLR | GGTTCCACTAGTTTAGAGTCTTGCT CTCACGTTCAGAC | GFP11CAT2 construction |
| 15 | Cat2promR | tccctctagaattgtacaAGGGTTTACCTTGTAAGGAT | CAT2 construction |
| 16 | Cat2prom F | AGATTGAATTGGATCATATA | CAT2 construction |
| 17 | CAT2F | gggg aca agt ttg tac aaa aaa gca ggc ttc ATGGATCCTTACAAGTATCGTCCAGC | CAT2 construction |
| 18 | CAT2RN | gggg acc ac ttt gta caa gaa agc tgg gtc TTAGATGCTTGGTCTCACG | CAT2 construction |
| 19 | CAT2RWSQV | gggg acc ac ttt gta caa gaa agc tgg gtc TTAGAGTCTTGCTCTCACGTTCAGACGGCTT | CAT2 construction |
| 20 | CAT2RARL | gggg acc ac ttt gta caa gaa agc tgg gtc TTA CAC CTG AGA CCA GTA AGA GAT CC | CAT2 construction |
| 21 | PSI-3UTR-F | ccctactgtacaACGAGTGAAAGAATTCTTGATTGG | CAT2 construction |
| 22 | PSI-3UTR-R | ccctactgtacaCTGAAATCTTGATCTCTGTTACTG | CAT2 construction |
| 23 | WSQV-3UTR- | cctagtctaga GCAGACTAAAAATCACGTCTT | CAT2 construction |
| 24 | ARL-3UTR-R | cctagtctagaTGTCAAAACCAAGTTTATTTTC | CAT2 construction |
| 25 | *CAT2*-F | CGAGGTATGACCAGGTTCGT | RT-PCR |
| 26 | *CAT2*-R | GATGCTTGGTCTCACGTTCA | RT-PCR |
| 27 | *CAT2-1*R | GACTTATCAGCCTGAGACCA | RT-PCR |
| 28 | *CAT2-2*R | GAACGAGACAATGACAGTAACAGA | RT-PCR |

**Table S2.** Table of actual catalase activities. Values unit : (µmol H_2_O_2_ mg^-1^ protein min^-1^), SD; short-days, LD; long-days. Data presented are mean values. SE; standard error

|  | SD | LD |
| --- | --- | --- |
| Wild type | 171±20.3 | 198±17.1 |
| *Cat2-1* | 37±6.8 | 44±4.4 |
| PSI1 | 148±16.7 | 178±19.9 |
| WSQV1 | 167±16.1 | 173±15.8 |
| Wild type | 60±4.6 | 84±0.6 |
| *Cat2-1* | 19±1.0 | 31±1.3 |
| PSI2 | 59±2.9 | 82±2.1 |
| WSQV2 | 60±2.4 | 78±3.7 |
| ARL1 | 53±2.8 | 47±2.7 |
| ARL2 | 47±4.8 | 53±1.0 |
| Wild type | 111±1.9 | 81±2.4 |
| *Cat2-1* | 21±0.8 | 30±1.1 |
| PSI3 | 107±0.01 | 73±2.2 |
| WSQV3 | 112±0.04 | 76±2.8 |

**Table S3.** Table of number of protoplasts observed with fluorescence in each organelle.

| Plasmid | Peroxisomes | Cytoplasm | Nucleus | Unknown |
| --- | --- | --- | --- | --- |
| Positive control | 14 | 10 | 15 | - |
| CAT2-PSI-sfGFP11 | 23 | 0 | 13 | - |
| CAT2-WSQV-sfGFP11 | 20 | 0 | 0 | - |
| CAT2-ARL-sfGFP11 | 0 | 0 | 0 | 2 |
